# Potential Health Benefits Associated with Lunasin Concentration in Dietary Supplements and Lunasin-Enriched Soy Extract

**DOI:** 10.3390/nu13051618

**Published:** 2021-05-12

**Authors:** Elvira Gonzalez de Mejia, Erick Damian Castañeda-Reyes, Luis Mojica, Vermont Dia, Hui Wang, Toni Wang, Lawrence A. Johnson

**Affiliations:** 1 Department of Food Science and Human Nutrition, University of Illinois at Urbana-Champaign, Urbana, IL 61801, USA; edreyes@illinois.edu (E.D.C.-R.); lmojica@ciatej.mx (L.M.); vdia@utk.edu (V.D.); 2 Tecnología Alimentaria, Centro de Investigación y Asistencia en Tecnología y Diseño del Estado de Jalisco, A. C., CIATEJ, Guadalajara 44270, Mexico; 3 Center for Crops Utilization Research, Iowa State University, Ames, IA 50011, USA; huiwang@iastate.edu (H.W.); twang46@utk.edu (T.W.); ljohnson@iastate.edu (L.A.J.)

**Keywords:** lunasin-rich supplements, ultrafiltration, lunasin-enriched soy extract, antioxidant potential, anti-melanoma activity

## Abstract

Lunasin has demonstrated antioxidative, anti-inflammatory, and chemopreventive properties. The objectives were to evaluate the concentration of lunasin in different lunasin-based commercial dietary supplements, to produce a lunasin-enriched soy extract (LESE) using a two-step pilot-plant-based ultrafiltration process, and to evaluate their biological potential in vitro. LESE was produced using 30 and 1 kDa membranes in a custom-made ultrafiltration skid. Lunasin was quantified in eight products and LESE. Lunasin concentrations of the lunasin-based products ranged from 9.2 ± 0.6 to 25.7 ± 1.1 mg lunasin/g protein. The LESE extract contained 58.2 mg lunasin/g protein, up to 6.3-fold higher lunasin enrichment than lunasin-based dietary supplements. Antioxidant capacity ranged from 121.5 mmol Trolox equivalents (TE)/g in Now^®^ Kids to 354.4 mmol TE/g in LESE. Histone acetyltransferase (HAT) inhibition ranged from 5.3% on Soy Sentials^®^ to 38.3% on synthetic lunasin. ORAC and lunasin concentrations were positively correlated, and HAT and lunasin concentrations were negatively correlated (*p* < 0.05). Melanoma B16-F10 and A375 cells treated with lunasin showed dose-dependent inhibitory potential (IC_50_ equivalent to 330 and 370 μM lunasin, respectively). Lunasin showed protein kinase B expression (57 ± 14%) compared to the control (100%) in B16-F10. Lunasin concentration found in commercial products and lunasin-enriched soy extract could exert benefits to consumers.

## 1. Introduction

Lunasin is a forty-three amino acid peptide originally isolated from soybeans. It features a unique amino acid sequence containing an arginine-glycine-aspartic acid cell adhesion motif and a polyaspartic acid tail on its carboxylic acid end [1,2,3,4,5,6,7,8,9,10,11,12]. The internalization of lunasin in macrophages is primarily mediated by endocytic mechanisms that involve integrin signaling, clathrin-coated structures, and micropinosomes. The aggregation of the clathrin-coated structures and the punctate localization of lunasin at the intracellular sites could indicate lunasin endocytosis and internalization into the nucleus via nucleolar sequestration [13].

Lunasin exhibits different biological and chemopreventive properties including anti-inflammatory, anticarcinogenic, antioxidant and immune-modulating properties, anti-atherosclerosis, and osteoclastogenesis inhibition potential [1,2,3,4,5,6,7,8,9,10,11,12,13].

The reported anti-inflammatory property is attributed to its capability to inhibit the translocation of the transcription factor NF-κB [14] while the antioxidant property is attributed to its potential to scavenge peroxy radicals and block Fenton reactions by chelating ferrous ions, thereby leading to protection of DNA from oxidative damage [15]. Besides, lunasin can potentiate the chemopreventive effects of other compounds, such as aspirin and anacardic acid [16]. Lunasin has shown anticancer properties in vivo against breast [17], skin [18], and colon cancers [19]. Its potential application in cancer immunotherapy has been explored, through in vitro and in vivo lymphoma models, showing that the combination of lunasin with cytokines IL-12 and/or IL-2 has higher tumoricidal activity than without [7]. Furthermore, lunasin can act as a vaccine adjuvant in activating dendritic cell function in models of lymphoma [20]. Besides, lunasin inhibits histone H3 and H4 acetylation when using a histone acetyltransferase assay [21].

Because of these reported health benefits, increasing numbers of lunasin-based dietary supplements are now available on the market. Patented products containing lunasin have been commercialized in the US market for their claimed ability to lower cholesterol and low-density lipoprotein cholesterols [22] and their claimed ability to increase leptin and adiponectin plasma levels indicating a possible role in preventing obesity [23].

Gonzalez de Mejia et al. [24] analyzed lunasin in commercial soy protein isolates and isoflavone products. Hernandez-Ledesma et al. [25] also evaluated lunasin and Bowman-Birk inhibitors in US commercially available soymilk and soy-based infant formulas using western blot. In addition, Cavazos et al. [26] analyzed lunasin concentrations in organic soymilk, soy protein shakes, and infant formulas using an enzyme-linked immunosorbent assay (ELISA). On the other hand, there are patented lunasin purification methods (WO2011060181A1), this patent includes methods for an extraction solution, multiple process steps for lunasin purification including size-based filtration, charge-based filtration, and hydrophobicity-based filtration techniques [27]. However, no studies on lunasin concentration and its biological potential in lunasin-based commercial dietary supplements have been reported. The hypothesis of this research is that it is possible to produce, at a pilot-plant level, a lunasin-enriched soy extract (LESE) with a higher concentration of lunasin and higher bioactivity than lunasin-based commercial products. The objectives of the present study were to evaluate the concentration of lunasin in different lunasin-based commercial dietary supplements, to produce a lunasin-enriched soy extract using a two-step pilot-plant-based ultrafiltration process, and to evaluate their biological potential.

## 2. Materials and Methods

### 2.1. Materials

Defatted soybean flour was provided by Archer-Daniels-Midland Company (Decatur, IL, USA). Lunasin rabbit polyclonal antibody was provided by Dr. Ben O. de Lumen (University of California, Berkeley, CA, USA). All other chemicals were purchased from Sigma-Aldrich (St. Louis, MO, USA) unless otherwise specified.

### 2.2. Lunasin-Based Commercial Dietary Supplement Samples

Lunasin-based commercial samples were purchased online. Eight samples were purchased: (1) Soy Sentials^®^, (2) Slimplicity^®^, (3) Now^®^, (4) Provantage^®^, (5) LunaRich X™ capsule containing a pure concentrated form of lunasin, (6) GlucAffect^®^, (7) Now^®^ kids-vanilla, and (8) Now^®^ kids-chocolate. Ingredients for each dietary supplement are presented in Table 1.

### 2.3. Pilot-Plant Production of Lunasin-Enriched Soy Extract

Production of lunasin-enriched soy extract was performed in the pilot plant of the Center for Crops Utilization Research, Iowa State University (Ames, IA, USA). Defatted soy flour was suspended in deionized water in a 1:10 ratio and mixed for 90 min at room temperature. After that, the mixture was centrifuged with a Centrisys horizontal decanter (Model no. CS10-4 3PH, Kenosha, WI, USA) at 4255 rpm bowl speed, 2 rpm differential solid scroll speed, with 11.5 L/min (LPM) feed speed. The resulting supernatant was subjected to two-stage ultrafiltration using membranes with 30 kDa and 1 kDa molecular weight cut-offs obtained from Sepro Membranes, Inc. (Oceanside, CA, USA). The pilot-plant ultrafiltration system was a custom-made skid manufactured by MP&C, Inc. (Edgar, WI, USA). The ultrafiltration was carried out using the 30 kDa cartridge under the following conditions: a feed rate of 13 LPM, a recirculation pump outlet pressure of 30 psi, and a permeate flow rate of 1.6 LPM by adjusting the retentate/permeate ratio control valve and the recirculation pump and the high-pressure pump. The permeate containing molecules with molecular weight <30 kDa were collected and further subjected to another ultrafiltration step using 1 kDa molecular weight cut-off membrane with the following conditions: a feed rate of 10 LPM, a recirculation pump outlet pressure of 100 psi, and a permeate flow rate of 1.2 LPM by adjusting the retentate/permeate ratio control valve and the recirculation pump and the high-pressure pump. The concentrated retentate was collected and freeze-dried as the final enriched lunasin extract.

### 2.4. Analysis of Lunasin during Pilot-Plant Based Production of Lunasin-Enriched Soy Extract

Samples of the decanter supernatant, the retentates and permeates from both 30 and 1 kDa membrane treatments were analyzed for lunasin using sodium dodecyl sulfate-polyacrylamide gel electrophoresis (SDS-PAGE) and western blot analysis as previously reported [1].

### 2.5. Lunasin Purification Anion Exchange Chromatography

Lunasin extract (LE) was obtained as reported previously [26] with slight changes. Briefly, freeze-dried lunasin enriched soy extract (40% *w*/*v*) was solubilized in 50 mL distilled water, centrifuged twice at 12,000× *g* for 10 min, and filtered through a 0.45 µm filter. A HiTrap Q HP (GE Healthcare Bio-Sciences, Uppsala, Sweden) column was used coupled with a HiPrepp 26/10 desalting pre-column (GE Healthcare Bio-Sciences, Uppsala, Sweden). Unbound proteins eluted with Tris-HCl 20 mM, pH 7.4 at a 1 mL/min flow rate. Bound proteins eluted using 0.4 M NaCl. The LE sample was desalted using ultrafiltration through a 1 kDa disc and freeze-dried for in vitro analysis.

### 2.6. Protein Concentration Measurement by Detergent-Compatible (DC) Protein Assay

The protein concentration of lunasin-based commercial products and samples from ultrafiltration production of lunasin-enriched flour were determined by microplate DC protein assay (Bio-Rad Laboratories, Hercules, CA, USA) as previously reported [1] and calculated using a bovine serum albumin (BSA) standard curve (y = 0.0003x + 0.0209, R^2^ = 0.99).

### 2.7. Enzyme-Linked Immunosorbent Assay

Lunasin concentrations of lunasin-based commercial products and samples from ultrafiltration production of lunasin-enriched soy extract were determined by ELISA as previously reported [1] and calculated using a synthetic lunasin standard curve (y = 0.0076x − 0.1902, R^2^ = 0.96).

### 2.8. Antioxidant Capacity

The antioxidant capacities of all the samples were measured by the oxygen radical absorbance capacity (ORAC) assay as previously described [28] and the results were expressed as mmol Trolox equivalents (TE)/g and calculated using the generated Trolox standard curve (y = 0.1150x − 3.17, R^2^ = 0.98).

### 2.9. Histone Acetyltransferase (HAT) Inhibitory Screening Assay

HAT inhibition assay was performed according to the manufacturer protocol (Cayman Chemicals, Ann Arbor, MI, USA). The assay was conducted as follows: 15 µL of assay buffer, 5 µL of acetyl CoA, 10 µL of diluted HAT/pCAF, and 5 µL of diluent or samples (final concentration of 100 µg protein/mL for samples and 10 µM for synthetic lunasin) were added to wells in triplicate. The reactions were initiated by adding 20 µL of HAT peptide except for background wells and incubated in a shaker for 5 min at room temperature. After incubation, the reaction was stopped by adding 50 µL of HAT stop reagent to all wells and 20 µL of HAT peptide was added to the background wells. After which, 100 µL of HAT developer was added to each well followed by incubating for 20 min at room temperature. The plates were read using a Synergy 2 microplate reader (Winooski, VT, USA) at 340/30 nm excitation wavelength and 440/40 nm emission wavelength. Inhibition of HAT was calculated based on the total HAT initial activity well (treated with diluent only).

### 2.10. Cell Cytotoxicity

B16-F10 mouse melanoma (ATCC^®^ CRL6475™) and A-375 human melanoma ATCC^®^ CRL-1619™) were purchased from American Type Culture Collection (Manassas, VA, USA). Cells were inoculated in 96-well plates at a confluence of 1 × 10^5^ cells per well and treated with different LE concentrations (0.158 ng/mL to 3.16 mg/mL) for 24 h. Cells were maintained at 37 °C, 5% CO_2_, and 95% air. Cell viability was measured using CellTiter 96^®^ AQ_ueous_ One Solution (Promega Corporation, Madison, WI, USA) according to manufacturer protocol.

### 2.11. Protein Kinase B (Akt) Pathway Expression

Cells were treated with lunasin half-maximum inhibitory concentration (IC_50_) for 24 h, and then cell lysates were produced. The protein concentration was quantified with Bio-Rad DC protein assay. Based on preliminary results obtained using Mitogen-Activated Protein Kinases (MAPK) (ab211061; Abcam, Cambridge, MA, USA) array (data not shown), Protein kinase B (Akt) (AAH-AK T-1-8; Ray Biotech, Norcross, GA, USA) was focused on using a protein concentration of 300 µg of protein/mL. The epitopes for AKT array are shown in Table 2.

### 2.12. Statistical Analysis

All analyses were performed in at least two independent replicates with two replicates. Data were analyzed using the proc GLM command in SAS software version 9.4. Statistical significance was reported at *p* < 0.05 and Tukey posthoc test was applied. GraphPad Prism 8 was used for IC_50_ analysis. AKT membranes were analyzed using a *t*-test.

## 3. Results and Discussion

### 3.1. Analysis of Lunasin Concentrations in Lunasin-Based Commercial Dietary Supplements

**Figure 1:**
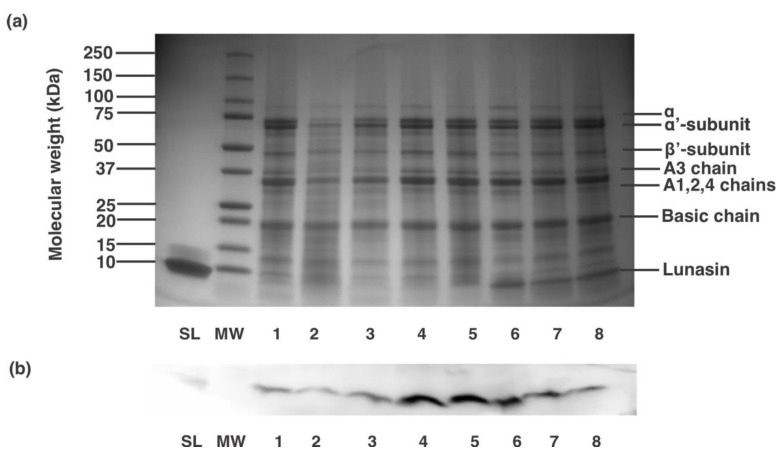
Analysis of commercial lunasin-based dietary supplements. (**a**) SDS-PAGE protein electrophoretic profile and (**b**) Western blot analysis of different lunasin-based products. Lanes SL and MW are synthetic lunasin and molecular weight standard, respectively. Samples were used at a concentration of 100 µg protein/mL and coded as 1: Soy Sentials^®^, 2: Slimplicity^®^, 3: Now^®^ dietary supplement, 4: Provantage^®^, 5: LunaRich X™, 6: GlucAffect^®^, 7: Now^®^ kids-vanilla and 8: Now^®^ kids-chocolate.

Table 1 presents lunasin concentrations while Figure 1 shows the protein profile (a) and western blot analysis (b) of different commercial lunasin-based dietary supplements. On a serving basis, LunaRich X™ capsule had the lowest lunasin concentration, 0.2 ± 0.0 mg lunasin/serving (41.0 ± 2.1 mg lunasin/100 g powder), while Now^®^ kids-vanilla presented the highest lunasin concentration, 7.6 ± 0.3 mg lunasin/serving (26.2 ± 1.1 mg lunasin/100 g powder) (Table 1). Figure 1a shows that all the lunasin-based products contained mixtures of different soy proteins, indicating a simple extraction and/or purification steps during preparation. The protein profiles of the eight products contain the major proteins found in soybeans as previously reported [29]. Figure 1b confirms the presence of lunasin in the commercial products as they show a positive reactivity towards lunasin rabbit polyclonal antibody. Studies on the concentration of lunasin in commercial soy products have been previously reported. No studies, however, evaluated lunasin concentrations in lunasin-based dietary supplements.

Cavazos et al. [26] reported that lunasin concentrations ranged from 1.6 to 22.2 mg lunasin per serving in different organic soymilks, soy protein shakes, and soy infant formulas. Hernandez-Ledesma et al. [30] reported that different soymilks had lunasin concentrations of 10.7 to 18.9 mg/100 mL of milk, which can be translated from 25.7 to 45.4 mg lunasin per 240 mL serving. Our results showed that the lunasin concentrations of lunasin-based products were 0.2 ± 0.0 to 7.6 ± 0.3 mg lunasin/serving (240 mL), which typically falls within the standard lunasin concentrations that can be found in different soymilk and soy-based infant formulas. Table 1 shows the lunasin concentration expressed as mg lunasin/100 g product and mg lunasin/serving, as well as serving size and ingredients that can be found in each commercial lunasin-based dietary supplement. Previous in vitro studies have shown that as little as 10 µM of lunasin led to reducing pro-inflammatory markers TNF-α, IL-6, and IL-1β in lipopolysaccharide-induced macrophages [14,25]. Considering the low bioavailability of lunasin and a total blood volume of five L, approximately 250 mg of lunasin is needed to reach the 10 µM lunasin concentration. Based on the lunasin concentrations in different lunasin-based commercial dietary supplements, approximately 33 servings (Now^®^ Kids-Vanilla) to 1250 servings (LunaRich X™ capsule) would be needed to reach this concentration. These estimates assume that lunasin is 100% bioavailable. Previous studies on the bioavailability and digestibility of lunasin showed that 97% of lunasin is digested after pepsin-pancreatin digestion [31] and only 4.5% of the remaining lunasin can be absorbed [32] indicating that a larger number of servings of these products is needed in order to reach an effective concentration of 10 µM. Continuous intake of soy-based products, however, may lead to sustained lunasin concentrations in plasma, which might be responsible for health benefits associated with soy consumption.

### 3.2. Pilot-Plant Production of Lunasin-Enriched Soy Extract

**Figure 2:**
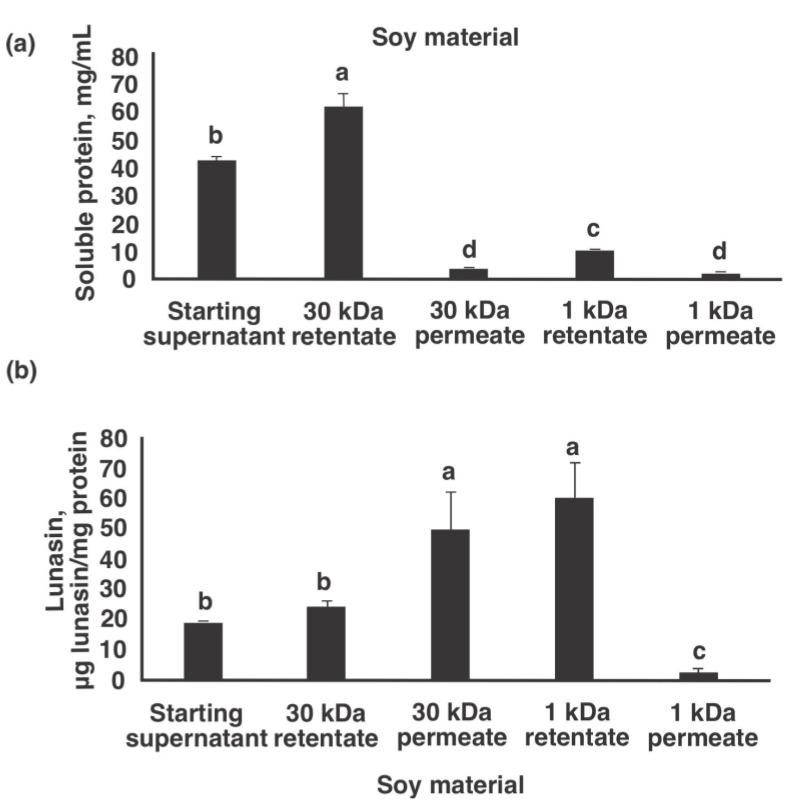
Soluble protein and lunasin analysis of different fractions obtained from two-step ultrafiltration pilot plant production of lunasin-enriched soy extract. (**a**) Soluble protein concentration as measured by protein DC assay, (**b**) Lunasin concentration as measured by ELISA. Different letters indicate significant differences among fractions (*p* < 0.05).

Figure 2a,b show the soluble protein and lunasin concentrations of soy materials at different stages of production of lunasin-enriched soy extract, respectively. The protein concentration of the starting supernatant after centrifuging the defatted soy flour:water mixture (1:10 ratio) was 42.6 mg/mL. After 30 kDa ultrafiltration, the retentate had an increased protein concentration to 62.5 mg/mL, which is expected as molecules <30 kDa passed through the 30 kDa membrane cartridge. The increase in total soluble protein can also be attributed to the concentration of the starting supernatant to 30 kDa retentate. On the other hand, the protein concentration of the 30 kDa permeate was 3.5 mg/mL, at least 12x lower than the starting supernatant. This could be attributed to dilution of this fraction as more water passed through the 30 kDa filter. Following ultrafiltering with the 30 kDa membrane, the permeate was collected and further concentrated using a 1 kDa membrane cartridge to remove low-MW compounds including sugars and salts. The protein concentration of the resulting 1 kDa retentate increased to 10.4 mg/mL, a 3-fold increase from the starting 30 kDa permeate. As expected, the protein concentration of the 1 kDa permeate was low at 2.1 mg/mL. The starting supernatant had a lunasin concentration of 18.2 ± 1.5 µg lunasin/mg protein, which was not statistically different (*p* > 0.05) from the lunasin concentration of the 30 kDa retentate (24.1 ± 2.8 µg lunasin/mg protein). On the other hand, the lunasin concentration of the 30 kDa permeate increased to 53.0 ± 11.0 µg lunasin/mg protein. The lunasin concentration of the 1 kDa retentate was similar to the 30 kDa permeate at 58.2 ± 11.4 µg lunasin/mg protein. This observation was expected as the 30 kDa permeate was only concentrated to obtain the 1 kDa retentate.

**Figure 3:**
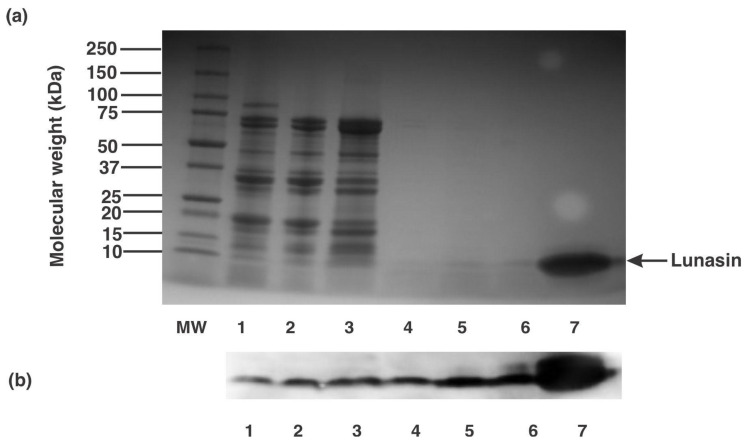
Protein profile and lunasin identification in pilot plant enrich fractions. (**a**) Protein electrophoretic profile and (**b**) Western blot analysis of different fractions from pilot plant production of lunasin-enriched soy extract. Lanes MW: molecular weight standard, 1: defatted soy flour:water mixture (1:10), 2: supernatant after centrifugation, 3: 30 kDa retentate, 4: 30 kDa permeate, 5: 1 kDa retentate, and 6,7: synthetic lunasin (10 µM).

From the starting concentration of 18.2 µg lunasin/mg protein in the decanted supernatant, the final 1 kDa retentate with lunasin concentration of 58.2 µg lunasin/mg protein presented a 3.2-fold enrichment of lunasin concentration. To further validate the presence of lunasin on the different soy fractions prepared during the two-step ultrafiltration process, the protein profile of each fraction was evaluated during the lunasin-enriched soy extract production. Figure 3a shows the electrophoretic gel profiles of the different fractions. Starting from the defatted soy flour:water mixture (lane 1), supernatant after centrifugation (lane 2), 30 kDa retentate (lane 3), 30 kDa permeate (lane 4), and 1 kDa retentate (lane 5), an approximately 5 kDa band was stained with SimplyBlue stain, which corresponded to the band of synthetic lunasin (lanes 6 and 7). The identities of lunasin in these fractions were further validated by the western blot profiles of the samples indicating positive reactivity towards lunasin rabbit polyclonal antibody (Figure 3b). Several reports have been done on the isolation and purification of lunasin from defatted soy flour. For instance, Seber et al. [33] used a combination of anion-exchange chromatography, reduction technique, ultrafiltration, and reverse-phase chromatography to obtain a >99% lunasin purity. While Park et al. [34] used a combination of ion-exchange chromatography and ultrafiltration techniques to obtain purified soy lunasin. Krishnan and Wang [35] reported a method to enrich lunasin based on the extraction of soybean flour with 30% ethanol followed by preferential precipitation of lunasin and protease inhibitors using calcium. This process yields 3.2 g of lunasin and protease inhibitors from 100 g of soybean flour. The present report is the first on the production of lunasin-enriched soy extract using a two-step ultrafiltration technique. The concentration of lunasin in our prepared LESE was 58.2 µg lunasin/mg protein; compared to the concentrations in commercial lunasin-enriched products (concentrations ranging from 9.2 to 25.7 µg lunasin/mg protein) this process enriched lunasin by 2.3 to 6.3-fold.

### 3.3. Antioxidant Capacity and Inhibition Potential of Histone Acetyltransferase (HAT)

**Figure 4:**
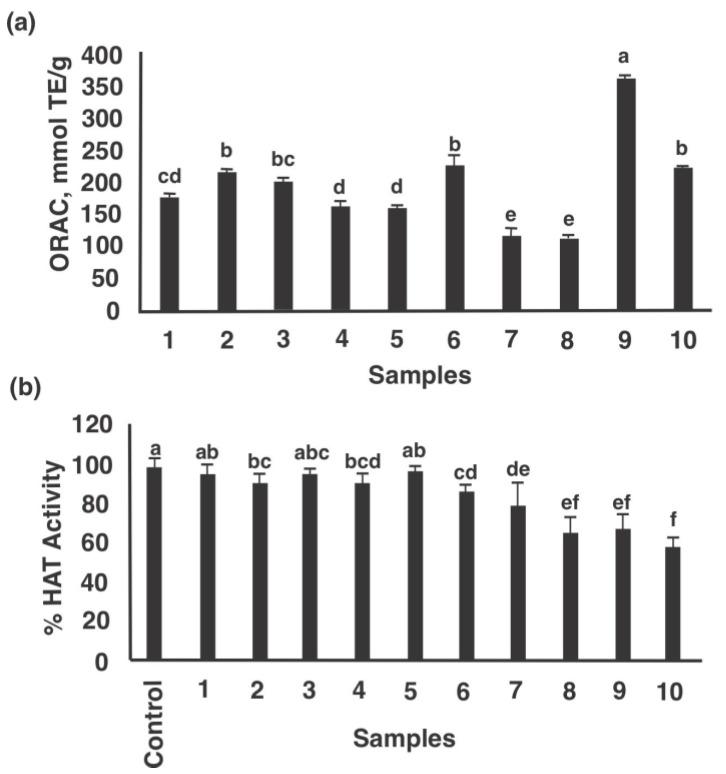
Biological potential of lunasin-based dietary supplements, lunasin-enriched soy extract, and synthetic lunasin. (**a**) Antioxidant capacity (mmol TE/g of product), (**b**) Histone acetyltransferase HAT (%) inhibitory potential. Samples were used at a concentration of 100 µg protein/mL and coded as 1: Soy Sentials^®^, 2: Slimplicity^®^, 3: Now^®^ dietary supplement, 4: Provantage^®^, 5: LunaRich X™, 6: GlucAffect^®^, 7: Now^®^ kids-vanilla and 8: Now^®^ kids-chocolate, 9: Freeze-dried lunasin enriched soy extract, and 10: Synthetic lunasin at 10 µM (*n* = 3). Different letters indicate significant differences among products (*p* < 0.05). Gallic acid (0.1 mg/mL) had an ORAC value of 220.3 ± 2.7 mmol TE/g, used for comparison purposes.

**Figure 5:**
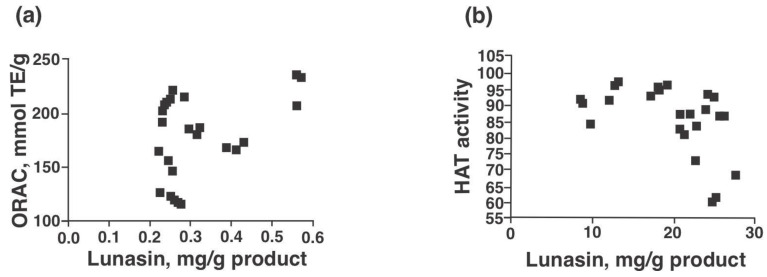
Lunasin content correlations with antioxidant capacity and HAT inhibitory potential. (**a**) Lunasin (mg/g product) positively correlated with ORAC values and (**b**) Lunasin (mg/g protein) negatively correlated with HAT activity. Samples were used at a concentration of 100 µg protein/mL; materials included: Soy Sentials^®^, Slimplicity^®^, Now^®^ dietary supplement, Provantage^®^, Lunarich X™, GlucAffect^®^, Now^®^ kids-vanilla and Now^®^ kids-chocolate (*n* = 3). Gallic acid (0.1 mg/mL) had an ORAC value of 220.3 ± 2.7 mmol TE/g, used for comparison purposes.

Figure 4a,b present the antioxidant capacity (ORAC) and HAT inhibitory potential of lunasin-based dietary supplements as well as lunasin-enriched soy extract and synthetic lunasin. The ORAC values of the samples ranged from 121.5 mmol TE/g product for Now^®^ kids-chocolate to 354.4 mmol TE/g for freeze-dried lunasin enriched soy extract (Figure 4a). HAT inhibition ranged from 5.3% for Soy Sentials^®^ to 38.3% for synthetic lunasin (10 µM) (Figure 4b). Significant positive correlation was found between ORAC and lunasin concentration of lunasin-based dietary supplements (Figure 5a, *p* = 0.046, Pearson r = 0.412) and significant negative correlation between HAT activity and lunasin concentration of lunasin-based dietary supplements (Figure 5b, *p* = 0.025, Pearson r = −0.456). Results indicate that the antioxidant capacity and the inhibition potential of HAT may be partially attributed to lunasin present in the different soy products. However, phytochemicals such as saponins, isoflavones, among others could also be present and exert an effect on the antioxidant capacity. Previous studies have shown the antioxidant potential of lunasin in different in vitro models including RAW 264.7 macrophages, HepG2 cells, and Caco-2 cells [6,15,29]. On the other hand, studies on the chemopreventive property of lunasin have focused on its ability to alter the histone acetylation/deacetylation process. Lunasin in vitro is an inhibitor of H4 acetylation by p300/cAMP response element-binding protein-associated factor depending on the position of lysine-acetylated [36].

### 3.4. Cell Cytotoxicity

**Figure 6:**
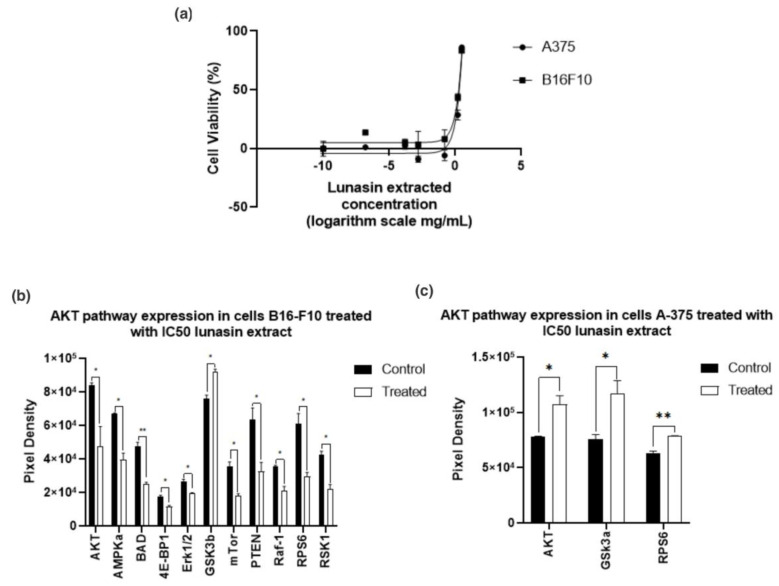
Melanoma cells treated with lunasin extract: (**a**) Cell viability: B16-F10 cell lines treated with different concentrations (0.158 ng/mL to 3.16 mg/mL). (**b**) Phosphorylated AKT pathway expression on cells B16-F10 after treatment with lunasin extract IC_50_ equivalent to 330 μM. (**c**) Phosphorylated AKT pathway expression on cells A-375 after treatment with lunasin extract IC50 equivalent to 370 μM. *p* values reflect the comparison of the control and the treated for each marker * *p* < 0.05, ** *p* < 0.005.

Figure 6a shows cell viability of B16-F10 and A-375 after LE treatment. Results showed that lunasin is effective in a concentration-dependent manner. Lunasin had an IC_50_ = 1.84 ± 0.06 mg/mL (equivalent to 330 μM) after 24 h on B16-F10 and IC_50_ = 2.03 ± 0.05 mg/mL (equivalent to 370 μM) on A-375. Shidal et al. [37], reported lunasin to be effective in A-375 cells after 36 h treatment with 100 μM lunasin; however, 50% cell inhibition was not achieved in this study. Lunasin was showed to be more effective in decreasing cell viability in A-375 melanoma cells after 24 h than oleuropein a bioactive compound from olive leaves, which needed 800 μM to decrease ~50% after 24 h [38].

In non-melanoma cell lines treated with lunasin the reported IC_50_ < 100 μM equivalent to <550 μg/mL after 24 h [31,39,40]. Jia et al. [41] treated synovial fibroblasts with lunasin up to 200 μM, after 48 h the IC_50_ was 153.3 ± 3.2 μM. After 24 h treatment, however, lunasin was not able to inhibit 50% of cell viability.

### 3.5. Protein Kinase B (Akt) Pathway Expression

The most common mutation in melanoma is located on the *BRAF* gene [42,43] which activates sequentially mitogen-activated protein kinase (MEK 1/2) and extracellular signal-regulated kinase (ERK1/2) leading to cell proliferation [42]. Previous studies reported that lunasin decreased phosphorylated (p-)ERK [19,44], we hypothesized that the cell growth inhibition could be due to an ERK inhibition. After 24 h treatment with IC_50_ in B16-F10 and A-375, the expression of p-AKT pathway (Figure 6) showed significant ERK inhibition (*p* < 0.05) for B16-F10. The inhibition of PRAS40 produced inhibition in mTOR, ribosomal protein S6 (RPS6), ribosomal protein S6 kinase (P70S6k), and eukaryotic translation initiation factor 4E binding protein 1 (4EBP1) interfering with cell growth and metabolism [45]. Lv et al. [45] reported that melanoma with high AKT activity also showed an increase in phosphorylated PRAS40.

Since p-glycogen synthase kinase 3 (GSK3) was significant in B16-F10 (*p* < 0.005) and A375 (*p* < 0.05) (Figure 6), our data suggested that lunasin promotes GSK3β inhibition through Ser9 phosphorylation. GSK3 functions in different cell processes including glycogen metabolism, proliferation, differentiation, motility, and survival. It could act as a tumor promoter or tumor suppressor [46,47,48]. GSK3β requires phosphorylation at Tyr216 for full activity, whereas, phosphorylation in Ser9 will result in an inactivation, which is the most important regulatory mechanism [46,48]. There is evidence that GSK3β inhibition limits motility and invasion of melanoma cells, which could lead to limit the metastatic behavior via N-cadherin signaling inhibition [49]. In an in vivo study where mice were fed with a soy powder mixture containing 52.6% of protein, 30.1% carbohydrates, and 5% lipids, inhibition on N-cadherin was shown [50]. Further studies to investigate any relationship between lunasin and N-cadherin signaling are needed.

As shown in Figure 6 and Table 2, AKT and RPS6 expressions were higher than the control for A-375 in 37% and 24%, respectively. A non-significant 24% overexpression in BAD compared with the control, however, was shown in the same cell line, which suggests that AKT and RPS6 overexpression, in this case, could not affect the cell growth.

## 4. Conclusions

The concentration of lunasin in different commercial lunasin-based dietary supplements was comparable to commercial soymilk and soy-based infant formulas. A simple two-step pilot-plant ultrafiltration process to generate lunasin-enriched soy extract was developed. This process is an alternative for enriching lunasin content in soybean products. Moreover, a significant positive correlation between ORAC and lunasin concentrations and a significant negative correlation between HAT activities and lunasin concentrations were found. Lunasin concentration in lunasin-based commercial dietary supplements and lunasin-enriched soy extract could increase the potential health benefits associated with soy consumption. Lunasin cytotoxicity in both melanoma cells was dose-dependent reaching a half-maximum inhibitory concentration at a concentration equivalent to 330 μM for B16-F10 and 370 μM for A375.

## Figures and Tables

**Table 1:** Lunasin and protein concentrations of different lunasin-based dietary supplements.

Product	Lunasin, mg/100 g Powder	Lunasin, mg/Serving	Protein *, g/Serving	Serving Size *, g (mL)	Other Ingredients
Soy Sentials^®^	31.1 ± 1.4 ^c^	6.8 ± 0.3 ^ab^	10	22 (240)	20 mg isoflavones, 2.5 g protective blend, LunaRich soy powder, red clover, wild Mexican yam, green tea extract (marketed as a protective supplement for women)
Slimplicity^®^	26.3 ± 1.8 ^cd^	7.1 ± 0.5 ^ab^	10	27 (240)	Conjugated linoleic acid, LunaRich soy powder, Advantra Z, L-carnitine, inulin, ChromeMate, CitriMax, Optizinc (marketed as meal replacement shake)
Now^®^	23.6 ± 0.6 ^d^	4.4 ± 0.1 ^c^	7	18.76 (240)	197 mg proprietary blend, LunaRich soy powder, vitamins and minerals
Provantage^®^	23.3 ± 1.1 ^d^	6.1 ± 0.3 ^b^	14	26 (240)	694 mg amino acid blend, 1220 mg performance blend, LunaRich soy powder, Tonalin, medium-chain triglycerides, creatine, CoQ10 (marketed as a dietary supplement for sports nutrition)
LunaRich X™	41.0 ± 2.1 ^b^	0.2 ± 0.0 ^d^	---	0.4 (capsule)	125 mg soy bioactive lunasin peptide (concentrated form of lunasin)
GlucAffect^®^	56.4 ± 0.6 ^a^	6.8 ± 0.1 ^ab^	5	12 (240)	15 mg pycnogenol, 2221 mg proprietary blend, LunaRich soy powder, omega-3-fish oils, Salacia extract, Glucohelp (marketed as a dietary supplement for maintaining healthy blood sugar)
Now^®^ for Kids (Vanilla)	26.2 ± 1.1 ^d^	7.6 ± 0.3 ^a^	5	29 (240)	408 mg proprietary blend, LunaRich soy powder, omega-3 fatty acid, phosphatidylserine, phosphatidylcholine, grape seed extract (marketed to help kids boost energy and mental performance)
Now^®^ for Kids (Chocolate)	25.1 ± 3.4 ^d^	7.3 ± 1.0 ^a^	5	29 (240)	408 mg proprietary blend, LunaRich soy powder, omega-3 fatty acid, phosphatidylserine, phosphatidylcholine, grape seed extract (marketed to help kids boost energy and mental performance)

* Taken from nutritional labels of the commercial products. Different letters per column indicate significant differences among products (*p* < 0.05).

**Table 2:** Protein kinase B (AKT) pathway protein expression of treated melanoma cells with IC_50_ compared to untreated control (100% expression).

Abbreviation	Full Name	Expression (%)	Epitope
B16-F10			
AKT	Protein B kinase	57	Ser473
AMPKa	AMP-activated protein kinase	59	Thr172
BAD	BCL2-associated agonist of cell death	51	Ser112
4E-BP1	4E-binding protein 1	68	Thr36
ERK 1/2	Extracellular signal-regulated kinase	72	ERK1 Thr202/Tyr204 ERK2 Tyr185/Tyr187
GSK3b	Glycogen synthase kinase 3b	119	Ser9
mTOR	Mammalian target of rapamycin	54	Ser2448
PTEN	Phosphatase and tensin homolog	56	Ser380
Raf-1	Rapidly accelerated fibrosarcoma-1	59	Ser301
RPS6	Ribosomal protein S6	52	Ser235/Ser236
RSK1	Ribosomal S6 kinase 1	50	Ser380
A-375			
Akt	Protein B kinase	137	Ser 473
GSK3a	Glycogen synthase kinase 3a	155	Ser21
RPS6	Ribosomal protein S6	124	Ser380

## Data Availability

Data is contained within this article.
